# Clinical decisions by the molecular tumor board on comprehensive genomic profiling tests in Japan: A retrospective observational study

**DOI:** 10.1002/cam4.5349

**Published:** 2022-10-17

**Authors:** Hidekazu Shirota, Keigo Komine, Masanobu Takahashi, Shin Takahashi, Eisaku Miyauchi, Hidetaka Niizuma, Hiroshi Tada, Muneaki Shimada, Tetsuya Niihori, Yoko Aoki, Ikuko Sugiyama, Maako Kawamura, Jun Yasuda, Shuhei Suzuki, Takeshi Iwaya, Motonobu Saito, Tsuyoshi Saito, Hiroyuki Shibata, Toru Furukawa, Chikashi Ishioka

**Affiliations:** ^1^ Department of Clinical Oncology Tohoku University Hospital Sendai Japan; ^2^ Department of Respiratory Medicine Tohoku University Graduate School of Medicine Sendai Japan; ^3^ Department of Pediatrics Tohoku University School of Medicine Sendai Japan; ^4^ Department of Breast and Endocrine Surgical Oncology Tohoku University Graduate School of Medicine Sendai Japan; ^5^ Department of Obstetrics and Gynecology Tohoku University School of Medicine Sendai Japan; ^6^ Department of Medical Genetics Tohoku University Graduate School of Medicine Sendai Japan; ^7^ Personalized Medicine Center Tohoku University Hospital Sendai Japan; ^8^ Division of Molecular Cellular Oncology Miyagi Cancer Center Research Institute Natori Japan; ^9^ Department of Clinical Oncology Yamagata University Faculty of Medicine Yamagata Japan; ^10^ Molecular Therapeutics Laboratory, Department of Surgery Iwate Medical University School of Medicine Morioka Japan; ^11^ Department of Gastrointestinal Tract Surgery Fukushima Medical University School of Medicine Fukushima Japan; ^12^ Department of Breast Surgery Japanese Red Cross Saitama Hospital Saitama Japan; ^13^ Department of Clinical Oncology, Graduate School of Medicine Akita University Akita Japan; ^14^ Department of Investigative Pathology Tohoku University Graduate School of Medicine Sendai Japan

**Keywords:** C‐CAT, comprehensive genomic profiling, molecular tumor Board, personalized medicine, solid cancer

## Abstract

**Background:**

A paradigm shift has occurred in cancer chemotherapy from tumor‐specific treatment with cytotoxic agents to personalized medicine with molecular‐targeted drugs. Thus, it is essential to identify genomic alterations and molecular features to recommend effective targeted molecular medicines regardless of the tumor site. Nevertheless, it takes considerable expertise to identify treatment targets from primary‐sequencing data in order to provide drug recommendations. The Molecular Tumor Board (MTB) denotes a platform that integrates clinical and molecular features for clinical decisions.

**Methods:**

This study retrospectively analyses all the cases of discussion and decision at the MTB in Tohoku University Hospital and summarizes genetic alterations and treatment recommendations.

**Results:**

The MTB discussed 1003 comprehensive genomic profiling (CGP) tests conducted in patients with solid cancer, and the resulting rate of assessing treatment recommendations was approximately 19%. Among hundreds of genes in the CGP test, only 30 genetic alterations or biomarkers were used to make treatment recommendations. The leading biomarkers that led to treatment recommendations were tumor mutational burden‐high (TMB‐H) (*n* = 32), *ERBB2* amplification (*n* = 24), *BRAF* V600E (*n* = 16), and *BRCA1/2* alterations (*n* = 32). Thyroid cancer accounted for most cancer cases for which treatment recommendation was provided (81.3%), followed by non‐small cell lung cancer (42.4%) and urologic cancer (31.3%). The number of tests performed for gastrointestinal cancers was high (*n* = 359); however, the treatment recommendations for the same were below average (13%).

**Conclusion:**

The results of this study may be used to simplify treatment recommendations from the CGP reports and help select patients for testing, thereby increasing the accuracy of personalized medicine.

## Graphical abstract

This study retrospectively analyzes all the cases of discussion and decision at the MTB in Tohoku University Hospital and summarizes genetic alterations and treatment recommendations. The findings of this study may be used to simplify treatment recommendations from the CGP reports and contribute to the selection of patients for testing, thereby augmenting the accuracy of personalized medicine.

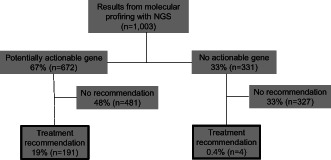

## 1 INTRODUCTION

Cancer is a disease marked by physiologically uncontrollable cellular proliferation due to numerous accumulated genetic alterations.^1^, ^2^ Lately, clinical oncology has shifted from organ‐specific treatment using cytotoxic agents to personalized medicine, in which treatment targets are identified per molecular profiling.^3^, ^4^ The two major reasons for this transition are the advent of next‐generation sequencing (NGS) and the emergence of various molecular‐targeted drugs.^5^, ^6^ Today, it is feasible to identify actionable molecular targets from genetic alterations in cancer and propose targeted molecular drugs irrespective of the tumor site.^7^ In Japan, the comprehensive genomic profiling (CGP) test is a part of routine medical care, and the Molecular Tumor Board (MTB) discusses all cases of solid cancer using this test.^8^

CGP testing, an NGS‐mediated targeted sequencing approach, is now covered by health insurance in Japan, making it feasible to recommend treatment based on genetic alterations. However, interpreting the complexity of tumor genetic and molecular features poses a challenge for clinicians to fully understand all the information and provide the most appropriate personalized treatment. This is because the one mutation–one drug recommendation that corresponds to genetic alterations in basic science may not have actual clinical therapeutic evidence. Thus, clinicians need to consider the differences in loci of genetic mutations, interactions with other genetic mutations, differences in the treatment evidence for various cancer types, patients' treatment history and condition, and ever‐evolving clinical trials' status. Such multiple factors and complexities led to the implementation of MTB. Therefore, discussions on the MTB are mandatory for the interpretation of CGP tests in the Japanese health insurance system.

This study reports the 2‐year experience of the MTB at a single Japanese facility that provides personalized treatment recommendations based on the CGP test results. In addition, this study compares and summarizes patient data from potentially actionable genes to actual treatment recommendations.

## 2 MATERIAL AND METHODS

### 2.1 Study design and setting

We conducted a retrospective observational study of CGP in patients with advanced solid tumors at Tohoku University Hospital and its affiliate hospitals, presenting detailed data on patient characteristics, genetic alteration lists, and treatment recommendations. All clinical cases underwent insurance‐covered CGP tests between September 2018 and January 2022 at seven facilities (Akita University Hospital, Iwate Medical University Hospital, Tohoku University Hospital, Yamagata University Hospital, Fukushima Medical University Hospital, Miyagi Cancer Center, and Saitama Red Cross Hospital), and the cases were discussed by the MTB (total: 106 times) at Tohoku University Hospital, with the remaining six facilities being affiliated. The Ethics Committee of Tohoku University Hospital approved this study. We obtained informed consent from all patients for using CPG data in clinical practice and in retrospective studies. In addition, patients were provided an opt‐out disclosure of details about specific studies.

### 2.2 Patients

Patients in this study were considered eligible for CGP testing if they had advanced solid tumors after the completion of standard therapy or were expected to complete the therapy. Patients were also considered eligible for pre‐treatment CGP testing for rare cancers or cancers of unknown primary origin (CUP) for which no standard of care was available. The attending physician ordered the test for cases deemed suitable for testing.

### 2.3 CGP test and C‐CAT report

The typically used CGP tests are FoundationOne CDx, FoundationOne liquid‐CDx genome profiling (F1 CDx and F1 liquid CDx; Chugai Pharmaceutical), and OncoGuide NCC Oncopanel System (NCC Oncopanel; Sysmex Corporation). These are Pharmaceuticals and Medical Devices Agency‐approved tests conducted using NGS‐based in vitro diagnostic devices and covered by the National Health Insurance in Japan. F1 CDx and NCC Oncopanel are designed for formalin‐fixed, paraffin‐embedded tumor tissue specimens, whereas F1 liquid CDx uses circulating cell‐free DNA isolated from plasma. F1 CDx is a tumor tissue‐based CGP assay. By sequencing 114 genes in normal and malignant tissues and determining gene differences, the NCC Oncopanel was created in Japan to detect somatic gene mutations. This enables simultaneous confirmation of germline mutations. In addition, F1 CDx and F1 liquid CDx apply NGS across 324 genes known to be drivers of solid tumors; all these platforms have been previously described and validated, and the methods have been explained elsewhere.^5^, ^9^ Moreover, NCC Oncopanel sequences 114 genes, covering all recommended treatments currently, as described elsewhere.^10^ Furthermore, F1 CDx and F1 liquid CDx are annotated and curated by Foundation Medicine, Inc. from proprietary databases, including public data, and report genetic variants and biomarkers considered pathological.

The Ministry of Health, Labor, and Welfare established the Center for Cancer Genomics and Advanced Therapeutics (C‐CAT) to provide information about cancer genome medicine under the health insurance system and devised a framework for recommending treatment based on a specific cancer knowledge database (CKDB). The C‐CAT comments on genetic alterations or biomarkers described in each patient's tumor profiling test report, referencing the CKDB to generate a C‐CAT report that is made available to the designated hospitals.^11^, ^12^ The MTB must use this report as the basis for treatment recommendations within the framework of the health insurance system.

### 2.4 MTB and treatment recommendation

**FIGURE 1:**
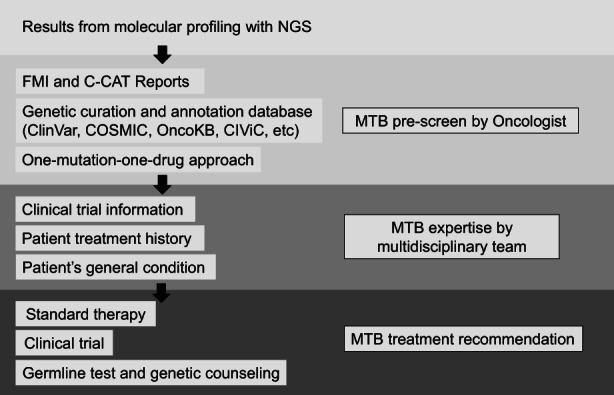
The critical step from comprehensive genomic profiling reports to Molecular Tumor Board recommendations.

The Japanese public insurance mandates discussing cases in the MTB before the attending physician explains the outcome to the patients. All case results were discussed at a weekly MTB meeting at our facility, which is attended by at least 10 oncology specialists. The represented specialties include gastroenterology, breast specialists, urology, gynecology, and pediatrics. Geneticists, genetic consultants, bioinformaticians, and other data sequencing experts also participate in such meetings. Although participation by the attending physicians is mandatory, more than 50 physicians from other institutions also participate. Figure 1 shows the process of arriving at an evaluation of a recommended treatment plan at the MTB meeting. We reviewed and discussed reports from the company (FMI and NCC), a report from C‐CAT, and our comprehensive report as a prescreening. The comprehensive report cited ClinVar,^13^ OncoKB,^14^ ToMMo,^15^ gnomAD,^16^ COSMIC,^17^ and CIViC.^18^ At the MTB meeting, the attending physicians presented the patients' medical history and general condition and discussed the possibility of treatment recommendations appropriate to patients and participation in a clinical trial. Notably, all treatment recommendations were accompanied by a level of evidence. In addition, actionable genetic alterations were categorized into levels of evidence based on the C‐CAT report and other databases as follows: level A, there is a Japan‐ or FDA‐approved drug for the cancer type; level B, there is a consensus among experts, supported by statistically credible clinical trials and meta‐analyses; level C, there is a Japan‐ or FDA‐approved drug for other cancers; level D, case reports demonstrated efficacy independent of cancer type; level E, pre‐clinical stage; and level F, genetic changes are known to be involved in cancer. Treatment recommendations at the MTB were made for cases with genetic alterations at level D or higher.

## 3 RESULTS

### 3.1 Patient characteristics and the CGP test

Table 1 lists the characteristics of 1003 patients [median age, 61 (range, 0–88) years] who underwent CGP testing. All cases successfully underwent insurance‐covered CGP tests from September 2018 to January 2022 at eight facilities in the northeast Japanese areas and were discussed at the MTB (106 times) in Tohoku University Hospital. All patients had advanced solid tumors. The attending physicians selected one of three CGP tests (F1 CDx, NCC Oncopanel, and F1 liquid CDx); Table 1 shows the number of each test. F1 CDx was used for 914 patients, while NCC Oncopanel and F1 liquid CDx were used for 25 and 64 patients. At our institution, 90% of cases undergo F1 CDx as a larger number of genes can be sequenced using this test. Thus, the cancer characteristics can be more closely investigated. In contrast, the NCC Oncopanel does not require confirmatory testing for patients with suspected hereditary tumors because the germline is sequenced simultaneously. Table 1 also shows the number and percentage of each type of tumor. In 1003 patients, the most frequent type of cancer was colorectal cancer (13.3%), followed by breast cancer (10.0%), sarcomas (9.9%), pancreatic cancer (8.8%), biliary tract cancer (7.3%), ovarian cancer (6.2%), head and neck cancer (6%), prostate cancer (4.7%), and CUP (3.5%). All cases were assessed for appropriate NGS analysis at the MTB meeting.

**TABLE 1:** Characteristics of patients, CGP test, and type of cancer

Median (range) age		61 (0–88)
Sex	Male (%)	482 (48)
	Female (%)	521 (52)
CGP test	FoundationOne CDx (%)	914 (91)
	FoundationOne liquid CDx (%)	64 (6.4)
	NCC Oncopanel (%)	25 (2.5)
	total	1003

Type of Cancer	No.	%
Colorectal	133	13.3
Breast	100	10.0
Sarcoma	99	9.9
Pancreatic	88	8.8
Bile duct	73	7.3
Ovarian	62	6.2
Head and neck	60	6.0
Prostate	47	4.7
CUP	35	3.5
Non‐small cell lung	33	3.3
Brain	36	3.6
Endometrial	27	2.7
Gastric	24	2.4
Esophageal	22	2.2
Small intestine	19	1.9
Thyroid	16	1.6
Kidney, urinary tract	16	1.6
Melanoma	15	1.5
Cervical Ca.	13	1.3
Thymic ca.	13	1.3
Neuroendocrine	10	1.0
Other	62	6.2
total	1003	100.0

### 3.2 CGP test and treatment recommendations

**FIGURE 2:**
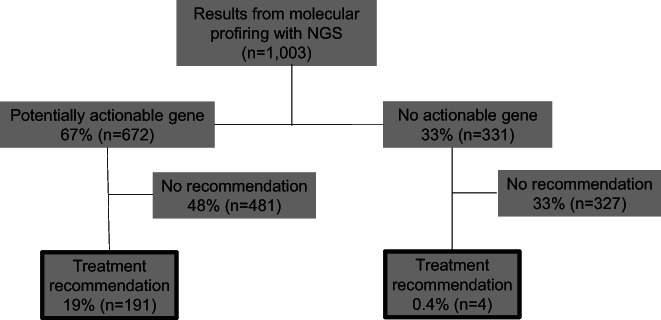
Consort diagram from next‐generation sequencing results to treatment recommendations.

Figure 1 explains the process from genetic alteration lists (CGP result) to actual treatment recommendations for genotype‐matched therapies. All genetic variants were assessed for pathogenicity and whether they could be associated with a therapeutic agent. In addition, genetic alterations for which treatment could be planned were judged as actionable genes and assigned a level of evidence. Actionable genetic alterations or biomarkers (with evidence of level D or higher) provided a 67% (672/1003) probability of recommending a drug from various databases (Figure 2). Of the 672 cases with potentially actionable genetic alterations, the MTB recommended the treatments for 191 cases (19% overall). These treatment recommendations were made based on alterations in 30 genes, and biomarkers in the MTB as listed in Table 2. In the classification by the level of evidence limited to the 30 genes, 42% (95/225) of treatment recommendations were made by level A or B, whereas 22% (121/544) were made by level C or D (Table 3). Treatment recommendations included drugs in clinical trials testing off‐label or new molecularly targeted drugs with therapeutic evidence. In contrast, drugs in phase 1 clinical trials with low evidence, such as drugs for TP53 and KRAS mutations, were not considered as treatment recommendations, but the information from clinical trials was made available to the patients. In four cases, the MTB identified the mutation as pathogenic and proposed treatment, even in patients for whom no treatment was recommended from the analysis‐provider's report (Figure 2); these biomarkers were *FGFR1* mutation, *FGFR2* amplification, *ALK* amplification, and *PIK3CA* mutation.

**TABLE 2:** List of genes and biomarkers that reached recommendations for treatments at MTB

*AKT1, ATM, ALK, BRAF non‐v600e, BRAF v600e, BRCA1, BRCA2, BRIP1, CDK12, CHEK2, CD274, EGFR, ERBB2 Amp., ERBB2, FGFR1 Amp., FGFR1‐3, IDH1, KIT, KRAS G12C, MET, MDM2 Amp., NTRK1‐3, PALB2, PIK3CA, PTEN, RET, ROS1, TSC1/2, TMB‐H, MSI‐H*

*Note*: Actionable genetic alterations were categorized into levels of evidence as follows: level A, there is a Japan‐ or FDA‐approved drug for the cancer type; level B, there is a consensus among experts, supported by statistically credible clinical trials and meta‐analyses; level C, there is a Japan‐ or FDA‐approved drug for other cancers; and level D, case reports demonstrated efficacy independent of cancer type.

**TABLE 3:** Number of evidence classification and treatment recommendations for the 30 genetic variants

Evidence level	Total No.	No. of recommend	Rate (%)
A	123	50	40.7
B	102	45	44.1
C	538	120	22.3
D	6	1	16.7

### 3.3 Treatment recommendations for each cancer type

Table 4 shows the number and implementation rates of recommendations by cancer type along with the number of changes in genes and biomarkers that led to recommendations for the treatment of each cancer type (including those without a recommendation). The rate of actual treatment recommendations was highest for thyroid cancer (81.3%), followed by non‐small lung cancer (42.4%), and kidney and urothelial carcinoma (31.3%). Thyroid cancers with *BRAF* mutations and *ROS1* fusion resulted in the highest frequency of recommendations. Non‐small cell lung cancers with *ERBB2* amplification, *MET* activation, *KRAS* G12C, *RET*, and *ROS1* fusion resulted in the second‐highest frequency of recommendations. In particular, some *EGFR* mutations were uncovered, although they were undetected in primary companion diagnostics. Moreover, breast cancer was associated with a high treatment recommendation rate (29%), but 50% of cases involved *PIK3CA* mutation for a clinical trial of the PIK3CA inhibitor. In addition, prostate cancer had a high rate of therapeutic target mutations, including *BRCA1/2*, *CDK12* mutations, and *ERBB2* amplification. Notably, 25% of prostate cancer cases were recommended for treatment. Furthermore, gastrointestinal cancers, such as colorectal cancer, gastric cancer, pancreatic cancer, and bile duct cancer, were related to low recommendation rates (<20%), wherein *ERBB2* amplification, *KRAS* G12C, and microsatellite instability (MSI)‐High (MSI‐H) resulted in recommendations commonly. Particularly, gastric (4.2%) and esophageal (0%) cancers were associated with a low rate of recommendation.

**TABLE 4:** Type of cancer and number of treatment recommendation and potentially actionable gene

Type of cancer	Total No.	No. of recommend	% of recommend	Potentially actionable gene (including genes that did not result with recommendation)
Colorectal	133	19	14.3	TMB‐H (5), ERBB2 amp (9), KRAS G12C (6), BRAF V600E (4), MSI‐H (2), FGFR2 fusion (2), FGFR3 fusion (1), MET (2), BRCA1 (1), BRCA2 (1)
Breast	100	29	29.0	PIK3CA (28), FGFR1 amp (13), FGFR2 fusion (1), AKT1 (4), BRCA1 (6), BRCA2 (10), ERBB2 amp (17), MET (2), NTRK3 fusion (2), TMB‐H (4), MSI‐H (1), PALB2 (3), PTEN (16), EGFR (1)
Sarcoma	99	11	11.1	TMB‐H (3), MSI‐H (2), MET (2), MDM2 amp (16), ERBB2 (2), BRAF V600E (2), FGFR2 (1), BRCA2 (1)
Pancreatic	88	7	8.0	BRCA1 (2), BRCA2 (4), KRAS G12C (2), MSI‐H (1), TMB‐H (1), MET (1), ERBB2 amp (1)
Bile duct	73	15	20.5	TMB‐H (8), ERBB2 amp (9), MET (3), BRAF V600E (1), MSI‐H (2), FGFR2 fusion (1), BRCA2 (2), EGFR (3)
Ovarian	62	15	24.2	BRCA1 (8), BRCA2 (2), FGFR1 amp (2), NTRK1 fusion (1), RET fusion (1), TMB‐H (2), ERBB2 amp (5)
Head and neck	60	13	21.7	BRCA2 (1), ERBB2 amp (4), FGFR3 (2), FGFR1 amp (3), TMB‐H (6), MET (1), EGFR (2)
Prostate	47	12	25.5	BRCA2 (7), BRCA1 (1), CDK12 (9), ERBB2 amp (3), BRAF V600E (1), TMB‐H (1)
CUP	35	9	25.7	TMB‐H (5), ERBB2 amp (3), BRCA1 (2), FGFR1 amp (2), BRCA2 (1), RET fusion (1), MSI‐H (1)
Non‐small cell lung	33	14	42.4	TMB‐H (7), ERBB2 amp (4), RET fusion (1), EGFR (5), MET amp or exon 14 skip (2), KRAS G12C (1), ROS1 fusion (1), BRCA1 (1), BRCA2 (1)
Brain	36	6	16.7	ALK amp (2), MET (3), BRAF V600E (2), KIT (2), IDH1 (8), ERBB2 amp (1)
Endometrial	27	4	14.8	TMB‐H (1), MSI‐H (1), ERBB2 amp (4), BRCA1 (4), FGFR2 (1), BRCA2 (1)
Gastric	24	1	4.2	ERBB2 amp (5), MSI‐H (1), TMB‐H (1)
Esophageal	22	0	0.0	TMB‐H (2), FGFR1 amp (1), BRCA1 (2), BRCA2 (1)
Small intestine	19	5	26.3	ERBB2 amp (1), KRAS G12C (1), MSI‐H (1), FGFR2 fusion (1), TMB‐H (1)
Thyroid	16	13	81.3	BRAF V600E (11)、RET fusion (2), MET (1), FGFR1 amp (1), EGFR (1), TMB‐H (1)
Kidney, urinary tract	16	5	31.3	TMB‐H (4), FGFR3 (3), FGFR2 (1), ROS1 fusion (1)
Melanoma	15	3	20.0	KIT (6), TMB‐H (2), BRAF V600E (2), MDM2 amp (3)
Cervical Ca.	13	0	0.0	ERBB2 amp (2)
Thymic ca.	13	1	7.7	TMB‐H (1)
Neuroendocrine	10	1	10.0	MSI‐H (1), TMB‐H (2), ROS1 fusion (1), FGFR1 amp (2), EGFR (2)
Other	62	12	19.4	
Total	1003	195	19.4	

### 3.4 Treatment recommendations based on each genetic biomarker

The overall incidence of genetic alterations, regardless of their pathogenicity, were listed for *TP53* (*n* = 662), *APC* (*n* = 278), *KRAS* (*n* = 244), and *CDKN2A* (*n* = 256), in Table S1. Excluding genetic alterations that were not therapeutic targets, such as TP53 and APC mutations, we focused only on the genetic alterations and biomarkers recommended for treatment in MTB, thereby narrowing the list to 30 biomarkers that were divided into the functional or signaling pathways (Tables 2 and 5). In addition, we categorized the number of recommended treatments for each biomarker and we determined whether they were standard therapy indications or clinical trial participation based on MTB discussions. Frequent actionable mutations comprised *PIK3CA* mutation (14%, *n* = 143); *BRCA1/2* mutation (6.3%, *n* = 63); *ERBB2* amplification (7.4%, *n* = 74), and tumor mutation burden‐high (TMB‐H) (5.5%, *n* = 55), as shown in Table 5. Biomarkers that led to treatment recommendations of the MTB were TMB‐H (*n* = 32), *ERBB2* amplification (*n* = 24), *BRCA1/2* mutation (*n* = 32), *BRAF* V600E (*n* = 16), and *FGFR1‐3* mutations or amplification (*n* = 30). Moreover, 195 patients (19%) with 216 biomarkers received actionable MTB recommendations. Overall, 64 cases were treatable with standard therapy, which is the treatment for this cancer covered by insurance in Japan, whereas 168 cases were recommended to undergo clinical trials (partially duplicates). The duplicated cases involved *BRCA1/2* mutations and were recommended to undergo either a platinum‐included chemotherapy regimen or a PARP inhibitor clinical trial. In addition to *BRCA1/2*, the newly recommended standard therapies include an immune checkpoint inhibitor for MSI‐H, trastuzumab regimen for *ERBB2* amplification, BRAF/MEK inhibitor for *BRAF* V600E, tyrosine kinase inhibitors for the fusion of *NTRK1,3*, *RET*, and *ROS1*, and everolimus for *PTEN* mutations or deletions in breast cancer. Table S2 presents the number of cancer types by major genetic alterations or biomarkers.

**TABLE 5:** Actionable gene alterations, biomarkers, and MTB recommendation

Signal pathway, function	gene or biomarker	Actionable	MTB recommend	Standard therapy	Clinical trials
PI3k/Akt/mTOR	*PIK3CA*	143	11		11
	*PTEN*	56	4	4	
	*TSC1/2*	19	3	1	2
	*AKT1*	13	1		1
RTK	*ERBB2 Amp*.	74	24	5	19
	*FGFR1 Amp*.	38	16		16
	*MET*	22	9	1	8
	*ERBB2*	19	3		3
	*FGFR1‐3*	18	14		14
	*EGFR*	17	3	2	1
	*KIT*	14	2		2
	*RET*	6	5	3	2
	*NTRK1‐3*	3	3	3	
	*ALK*	3	2		2
	*ROS1*	2	1	1	
RAS/RAF	*BRAF non‐v600e*	34	2		2
	*BRAF v600e*	22	16	2	14
	*KRAS G12C*	10	9		9
HRD	*BRCA2*	33	17	17	12
	*BRCA1*	30	15	15	4
	*ATM*	19	1		1
	*CDK12*	14	4		4
	*PALB2*	10	1		1
	*CHEK2*	6	1		1
	*BRIP1*	3	1		1
Immune (ICI)	*TMB‐H*	55	32		32
	*CD274*	15	3	3	
	*MSI‐H*	11	7	7	
other	*MDM2 Amp*.	46	4		4
	*IDH1*	14	2		2
	Total	769	216	64	168

## 4 DISCUSSION

This study demonstrates the real‐world data of new treatment recommendations based on the CGP test at a territorial single‐center MTB in Japan. In addition, MTB manages the selection and distribution of secondary findings; however, these findings are not included in the scope of this report. Overall, most patients with cancer were identified with pathogenic genetic alterations. Around two‐thirds had potentially actionable genetic alterations, which were clinically reported to be effective treatment at level D or higher. Nevertheless, only 19% of patients could receive new treatment recommendations after MTB discussions (Figure 2). Compared with previous reports, the percentage of patients with treatment recommendations ranged from 11% to 39%.^19^, ^20^, ^21^, ^22^, ^23^ Although the criteria for recommending treatment differ among MTBs and other countries have different clinical trial access, the results of our report appear to be reasonable.

For multiple reasons, some potential drugs raised by genetic alterations were not actually recommended. The rates of treatment recommendations for the actionable 30 biomarkers were 42% for those at level A or B and 22% for those at level C or D (Table 3). Moreover, 50 of 123 cases with biomarkers at level A were newly recommended for treatment. However, in 50% of such cases (*n* = 37), already known genetic alterations observed on companion diagnoses, including *BRAF* V600E, *EGFR* mutation, *ERBB2* amplification, and *BRCA1/2* mutation, were observed or no treatment recommendations were needed because the treatments were according to the standard therapy. For example, the immune checkpoint inhibitor was recommended for patients with TMB‐H but was treatable or already treated for non‐small cell lung cancer, gastric cancer, or head and neck cancer by standard therapy in the Japanese health insurance system. In some cases, despite an international therapeutic consensus with significant evidence levels, the immune checkpoint inhibitor has not yet been approved in Japan, and there are no clinical trials on the immune checkpoint inhibitors, such as those related to *PIK3CA* alterations in breast cancer. If there was a clinical trial indication at level C or D for a specific cancer type, such as amplification of *MDM2* or *FGFR1* or mutations of *AKT1*, *ATM*, *CDK12*, and *PTEN*, patients were considered as candidates for receiving treatment, although in most cases, no clinical trials were available. Moreover, there were cases in which patients' condition was poor to be eligible for a clinical trial. There were five main reasons why MTB did not recommend treatment, even in patients with potentially actionable genetic alterations. First, the genetic alteration was already identified and/or treated. Second, the patient received the therapy as standard treatment, permitted by insurance coverage. Third, the drug was inaccessible in Japan. Fourth, the patient's condition did not allow for the recommended treatment. Fifth, despite the evidence, the treatment was not recommended by the MTB. The reasons for each evidence level are summarized in Table S3.

The selection of cancer patients to be tested largely depends on the timing of testing defined under the Japanese health insurance system.^11^, ^12^ Indeed, the testing rate by cancer type differs from the general incidence rate of cancers. Only patients who have completed or are anticipated to complete their standard therapy as well as those with rare cancers or CUP for which no standard therapy is available are eligible for funding for the CGP test. As a result, even when they are in good health, patients with cancer who have multiple lines of standard therapy cannot usually undergo the test. Companion diagnoses, such as MSI, *ERBB2* staining, *EGFR*, *BRAF*, *ALK* fusion, and *BRCA1/2*, can be done before the standard treatment, which is another reason why the CGP test is not needed, as the drivers of lung cancer, colon cancer, and melanoma are already known to a certain extent. Hence, gastric and lung cancers tend to have low test rates, while rare cancers, such as sarcoma, tend to be more common. Importantly, real clinical data are available. The attending physician decides whether the CGP test needs to be conducted. Therefore, the percentage of genetic mutations by cancer type represents data from actual clinical practice; these data differ from those contained in cohorts, such as the TCGA databases. In general, treating physicians tend to test younger patients with the hope of identifying a therapeutic target. For example, in patients with lung cancer, the selection is based on young age, non‐smoking history, female gender, etc. These factors explain the selection bias for each cancer type and patient trait. NGS test recommendations should consider the current insurance reimbursement status in Japan, current evidence, and clinical trial results for each cancer type. In fact, the ESMO working group publishes recommendations for the use of NGS tests by cancer type.^24^ These recommendations are based on three levels of consideration: public health, the perspective of the academic clinical research center, and patient‐specific factors. Given the ESMO guidelines and our data, these guidelines would be useful in Japan.

In our facility, the most frequently proposed treatment was for thyroid cancer (81%), followed by non‐small cell lung cancer (42%). Breast cancer (29%), CUP (26%), and prostate cancer (26%) cases received recommendations relatively frequently. The *BRAF* mutation rates for thyroid cancer were high (63%, *n* = 11/16). The BRAF V600E mutation is responsible for a large percentage of thyroid cancer.^25^ However, because advanced recurrent thyroid cancer is relatively rare, there is no concomitant diagnosis of thyroid cancer or treatment targeting BRAF under the current Japanese insurance system. The only test for thyroid cancer in Japan involves genetic analysis using NGS. The authors hope that a concomitant diagnosis of the BRAF mutation will become available in the future. Many lung cancer cases had driver genes known before treatment by companion diagnosis, which resulted in small number of tests. Thus, CGP was performed primarily on patients identified as negative by the companion diagnosis. Remarkably, in some non‐small cell lung cancer cases, new undiscovered *EGFR* mutations or translocations were identified, and new treatment recommendations were provided in 40% of cases. In many patients with *PIK3CA* mutations, those with breast cancer were recommended for clinical trials. Moreover, CUP resulted in therapeutic targets in 25% of cases that should be recommended for testing. For gastrointestinal cancers, alterations or biomarkers in *ERBB2* amplification, *KRAS*, *BRAF*, *MSI‐H*, and *FGFR1‐3* were commonly detected in the colon and small intestine. As reported previously, it is challenging to find genetic targets to recommend a treatment to patients with stomach and esophageal cancer.^26^, ^27^ Nevertheless, the CGP test should be performed earlier for all patients; therefore, criteria should be defined to select cases and the timing of testing along with companion diagnoses for each organ‐specific cancer. Another example, patients with colon cancer of the microsatellite stable type with KRAS mutations have little chance of finding new therapeutic targets.

The CGP test sequences 114–324 cancer‐driver or ‐related genes. Indeed, we recommended only 30 different genes and biomarkers for treatment; realistically, that is all the biomarkers needed to provide a treatment recommendation. Then, genes or biomarkers were categorized by function and discussed based on the recommendation at our MTB as follows: (i) PI3K/AKT/mTOR signal: the activation of this signal pathway was recommended for clinical trials of *PIK3CA* inhibitor or standard therapy of the mTOR inhibitor, everolimus. In this category, the detection rate of genetic alterations was high, but the recommended treatment rate was low, and future treatment development would be anticipated. (ii) Receptor tyrosine kinases (RTK): The activation of RTKs, such as *EGFR, ERBB2, KIT*, and *PDGFR*, by mutation or translocation directly leads to tumor growth and is a clear therapeutic target; these mutations were detected in 216 of 1003 cases or around 20% of the cases. Of note, RTK is in the process of developing a treatment. In several cases already known by companion diagnosis with level A, new treatment recommendations were available for 82 patients. (iii) RAS/RAF pathway: the *KRAS* G12C mutation can be recommended for participation in clinical trials. Notably, *KRAS* mutations were the leading driver mutations in 244 patients (24%); of these, 10 were G12C mutations. Although MEK inhibitor is a candidate at level D, it would be ineffective with low evidence; thus, we are not recommending treatment currently. For *BRAF* alterations, treatment recommendations focused on treatable Class 1 mutations of V600E. This study newly identified 16 of 22 cases, and treatment recommendations were provided accordingly. Moreover, clinical trials were recommended in two cases due to *BRAF* alteration (*Non‐V600E*). For *BRAF* Class 2 and 3 mutations, MEK inhibitor is a candidate but would be ineffective with low evidence, and, thus, we are not recommending treatment. (iv) Homologous recombination deficiency (HRD): *BRCA1* and *BRCA2* mutations in the typical HRD gene were each 3% of the total. While half of the cases were already known to be breast or ovarian cancer cases, 32 cases were newly identified that also required confirmatory testing for secondary findings and were recommended for either a platinum‐containing regimen or a clinical trial of PARP inhibitor. Other HRD genes, including *ATM*, *PALB2*, and *CHEK2*, have little evidence and limited treatment recommendations. (v) Immune checkpoint inhibitor: This is recommended for patients with TMB‐H (>10 mut/Mb). This study included 55 patients with TMB‐H (5.5%); of these, 32 patients were recommended for ICI treatment. In Japan, ICI treatment for head and neck cancer, esophageal cancer, and gastric cancer is covered by insurance before CGP testing; thus, a TMB‐H diagnosis does not automatically indicate ICI treatment. For the CUP, TMB‐H comprised 14% of patients, which is almost consistent with the 12% of CUP reported in a previous study.^28^ Furthermore, MSI‐H occurred in 11 patients with various types of cancer.

All patients (574) were followed up to monitor their treatment courses. Of the 574 patients at our hospital, 119 (21%) were recommended for treatment. Of these, 42 (7.3%) followed the treatment suggestions and 77 (13.4%) chose other treatment options or the best supportive care. Despite treatment recommendations, in real‐life situations, not all patients will receive treatment. These data reflect real‐world medical care at our facility after CGP testing. Approximately 6% of cases at our facility participate in clinical trials. Some issues exist with the small number of clinical trials in Japan, such as challenges in using off‐label drugs and eligibility for participation in clinical trials. This study aimed to examine the MTB's assessment rate of treatment recommendation. However, it was not feasible to follow up on patients participating in clinical trials and their outcomes and overall survival because of the following: (i) The number of patients participating in clinical trials cannot be divulged due to company secrecy; (ii) few patients participated in clinical trials immediately after the test results, and it was possible only after several months had passed since the standard treatment was completed; and (iii) besides, more than half of the tests were conducted at other facilities where patients were not followed up.

Overall, this study retrospectively reviewed MTB treatment recommendations based on the CGP test, which can help simplify patient selection for genetic testing and targeted treatment by focusing on which mutations to be selected. MTB is under development in Japan. Therefore, treatment recommendations may differ relative to other institutions. In fact, even at our institution, the criteria vary depending on the timing of evidence and the presence or absence of clinical trials. Additional reports will allow further standardization and automation. The CGP test can provide significant confirmatory information to patients, although much of it is irrelevant to the actual treatment recommendations. However, a considerable part of the information is essential and can be used to handle VUS, confirm genomic instability, characterize cancer in CUP, propose a primary site, and estimate malignant grade. Initially, the one mutation–one drug approach was considered ineffective, especially in highly pretreated cancer patients. Even in the past 2 years, insurance indications in Japan have expanded, including drug approvals for multiple organs and increasing clinical trials. Furthermore, the ripple effect of implementing this test will broaden treatment options for patients with cancer and promote personalized medicine.

## AUTHOR CONTRIBUTIONS

**Hidekazu Shirota:** Conceptualization (lead); data curation (lead); formal analysis (lead); investigation (lead); methodology (lead); project administration (lead); writing – original draft (lead); writing – review and editing (lead). **Keigo Komine:** Conceptualization (supporting); data curation (supporting); formal analysis (supporting); investigation (supporting); project administration (supporting); supervision (equal); validation (supporting); writing – original draft (supporting). **Masanobu Takahashi:** Data curation (supporting); formal analysis (supporting); project administration (supporting); writing – original draft (supporting). **Takahashi Shin:** Data curation (supporting); validation (supporting); writing – original draft (supporting). **Eisaku Miyauchi:** Data curation (supporting); formal analysis (supporting); project administration (supporting); writing – original draft (supporting). **Hidetaka Niizuma:** Investigation (supporting); writing – original draft (supporting). **Hiroshi Tada:** Data curation (supporting); supervision (supporting); writing – original draft (supporting). **Muneaki Shimada:** Supervision (supporting); writing – original draft (supporting). **Tetsuya Niihori:** Validation (supporting); writing – original draft (supporting). **Yoko Aoki:** Supervision (supporting); writing – original draft (supporting). **Ikuko Sugiyama:** Formal analysis (supporting); methodology (supporting); project administration (supporting). **Maako Kawamura:** Formal analysis (supporting); methodology (supporting); project administration (supporting). **Jun Yasuda:** Project administration (supporting); supervision (supporting); writing – original draft (supporting). **Shuhei Suzuki:** Resources (supporting); writing – original draft (supporting). **Takeshi Iwaya:** Project administration (supporting); resources (supporting); writing – original draft (supporting). **Motonobu Saito:** Project administration (supporting); resources (supporting); writing – original draft (supporting). **Tsuyoshi Saito:** Project administration (supporting); resources (supporting); writing – original draft (supporting). **HIROYUKI SHIBATA:** Project administration (supporting); resources (supporting); writing – original draft (supporting). **Toru Furukawa:** Conceptualization (supporting); data curation (supporting); formal analysis (supporting); resources (supporting); supervision (equal); validation (supporting); visualization (supporting); writing – original draft (supporting); writing – review and editing (supporting). **Chikashi Ishioka:** Conceptualization (equal); funding acquisition (equal); investigation (equal); supervision (equal); validation (equal); visualization (equal); writing – original draft (equal); writing – review and editing (equal).

## CONFLICT OF INTEREST

Dr. Ishioka has received scholarship (incentive) endowments from Takeda, Daiichi‐Sankyo, Ono, Asahi‐Kasei Pharma, Taiho, and Chugai, as well as a research grant from Hitachi and Riken Genesis. All remaining authors have declared no conflict of interest.

## ETHICS STATEMENT

Approval of the research protocol by an Institutional Reviewer Board: IRB No. 2021–1‐250.

## DISCLOSURE

This research received no external funding.

## Supporting information


Tables S1–S3

## Data Availability

N/A
