# Supplementary material for: Clinical decisions by the molecular tumor board on comprehensive genomic profiling tests in Japan: A retrospective observational study
Source: Cancer Med. 2022 Oct 17;12(5):6170–81. doi: 10.1002/cam4.5349 (PMC10028111; doi:10.1002/cam4.5349)
Supplement: Supplementary file 1 — Tables S1–S3 [file CAM4-12-6170-s001.docx]

Supplementary Table S1. Top 10 genomic alterations in 1003 patients.

| Gene | Total No. | No. of Patients (Rate) |
| --- | --- | --- |
| TP53 | 662 | 594 (59%) |
| APC | 278 | 271 (27%) |
| CDKN2A | 256 | 245 (24%) |
| KRAS | 244 | 235 (23%) |
| KMT2D | 183 | 161 (16%) |
| NOTCH1 | 173 | 160 (16%) |
| PIK3CA | 168 | 145 (14%) |
| BRCA2 | 161 | 141 (14%) |
| CDKN2B | 156 | 155 (15%) |
| ARID1A | 130 | 114 (11%) |
| ATM | 116 | 108 (10%) |

| Supplementary Table S2. Actionable gene and Number of Cancer type | | |  |
| --- | --- | --- | --- |
| Biomarker | type of cancer (No.) |  |  |
| PIK3CA | Bile duct 8, Brain 3, Breast 29, Colorectal 22, CUP 6, Endometrial 12, Esophageal 7, Gastric 2, Head & Neck 14, NEC 1, Lung 2, Ovarian 14, Pancreatic 5, Prostate 6, Sarcoma 6, Small intestine 1, Cervical 3, Other 1 | | |
| ERBB2 Amp. | Bile duct 9, Brain 1, Breast 17, Colorectal 9, CUP 3, Endometrial 4, Gastric 5, Head & Neck 4, Ovarian 5, Pancreatic 1, Prostate 3, Sarcoma 2, Cervical 2, Small intestine 1, Other 8 | | |
| MET | Bile duct 4, Brain 3, Breast 2, Colorectal 2, CUP 1, Head & Neck 1, Lung 2, Pancreatic 1, Sarcoma 4, Thyroid 1, Other 1 | | |
| EGFR | lung 5, Bile duct 3, Breast 1, NEC 3, Melanoma 1, Head & Neck 2, Thyroid 1, Other 1 | | |
| KIT | GIST 3, Colorectal 3, Head & Neck 1, Melanoma 3, NEC 1, Prostate 1, Other 2 | | |
| RET | CUP 1, Lung 1, Ovarian 1, Prostate 1, Thyroid 2 | |  |
| FGFR1-3 | Breast 2, Colorectal 3, CUP 1, Endometrial 1, Head & Neck 3, Lung 1, Ovarian 1, Sarcoma 3, Urinary tract 2, Other 2 | | |
| NTRK1-3 | Breast 2, Ovarian 1 |  |  |
| ALK | Brain 2, Head & Neck 1 |  |  |
| ROS1 | Lung 1, Other 1 |  |  |
| BRAF V600E | Bile duct 1, Brain 2, Colon 4, Melanoma 2, Sarcoma 2, Thyroid 11 | | |
| KRAS G12C | Colorectal 6, Lung 1, Pancreatic 2, small intestine 1 | | |
| BRCA2 | Bile duct 2, Breast 10, Colon 1, CUP 1, Endometrial 1 Esophageal 1, Head & neck 1, Ovary 2, Pancreatic 4, Prostate 7, Sarcoma 1, other 2 | | |
| BRCA1 | Breast 7, Colon 1, CUP 2, Endometrial 4, Esophageal 2, Lung 1, Ovary 8, Pancreatic 4, Prostate 1 | | |
| TMB-H | Bile duct 8, Breast 3, Colorectal 4, CUP 5, Endometrial 1, Esophageal 1, Gastric 1, Head & Neck 6, melanoma 2, Lung 7, Ovarian 2, Pancreatic 1, Prostate 1, Sarcoma 4, Small intestine 2, Thymus 1, Other 5 | | |
| MSI-H | Bile duct 2, Breast 1, Colorectal 2, CUP 1, Endometrial 1, Gastric 1, Pancreatic 1, Sarcoma 2 | | |

| Supplementary Table S3. The number of reasons for no treatment recommendation | | | | |
| --- | --- | --- | --- | --- |
|  | Level A | Level B | Level C | Level D |
| The genetic alteration was already identified and/or treated. | 37 |  |  |  |
| It is in the standard therapy. | 1 | 15 |  |  |
| The drug is inaccessible in Japan. | 22 | 28 | 108 |  |
| Patient's condition does not allow for the recommended treatment. | 9 | 12 | 25 | 1 |
| Despite the evidence, the treatment was not recommended at the MTB. | 4 | 2 | 285 | 4 |
|  | 73 | 57 | 418 | 5 |
